# Co-designing community-based interventions to tackle antimicrobial resistance (AMR): what to include and why

**DOI:** 10.1186/s13104-023-06449-1

**Published:** 2023-10-24

**Authors:** Jessica Mitchell, Abriti Arjyal, Sushil Baral, Dani Barrington, Paul Cooke, Fariza Fieroze, Rumana Huque, Prudence Hamade, Helen Hawkings, Nichola Jones, Sophia Latham, Ayuska Parajuli, Md Badruddin Saify, Rebecca King, the CE4AMR network

**Affiliations:** 1 Nuffield Centre for International Health and Development, Faculty of Medicine, University of Leeds, Woodhouse, Leeds, LS2 9JT UK; 2 HERD International, Bhaisepati, Lalitpur, Nepal; 3 University of Western Australia, School of Population and Global Health, 35 Stirling Highway, Crawley, WA 6009 Australia; 4 Centre for World Cinema and Digital Cultures, Faculty of Arts and Humanities, University of Leeds, Woodhouse, Leeds, LS2 9JT UK; 5 ARK Foundation, Dhaka, 1212 Bangladesh; 6 Malaria Consortium, The Green House 244-254 Cambridge Heath Rd, London, EC2 9DA UK; 7 Department of Livestock and One Health, Institute of Veterinary and Ecological Sciences, University of Liverpool, Leahurst Campus, Chester High Road, Neston, CH64 7TE UK

**Keywords:** Antimicrobial resistance, Community, One health, Participatory approaches

## Abstract

Antimicrobial resistance (AMR) is a social and biological problem. Although resistance to antimicrobials is a natural phenomenon, many human behaviors are increasing the pressure on microbes to develop resistance which is resulting in many commonly used treatments becoming ineffective. These behaviors include unregulated use of antimicrobial medicines, pesticides and agricultural chemicals, the disposal of heavy metals and other pollutants into the environment, and human-induced climatic change. Addressing AMR thus calls for changes in the behaviors which drive resistance. Community engagement for antimicrobial resistance (CE4AMR) is an international and interdisciplinary network focused on tackling behavioural drivers of AMR at community level. Since 2019 this network has worked within Low-Middle Income Countries (LMICs), predominantly within Southeast Asia, to tackle behavioral drivers of AMR can be mitigated through bottom-up solutions championed by local people. This commentary presents seven *Key Concepts* identified from across the CE4AMR portfolio as integral to tackling AMR. We suggest it be used to guide future interventions aimed at addressing AMR via social, participatory, and behavior-change approaches.

## Introduction

Antimicrobial resistance (AMR) is the biological process by which microbes evolve to resist the effects of antimicrobial medicines [1]. Drug resistant infections are more difficult and costly to treat and can be lethal. 1.27 million human deaths were directly attributed to bacterial AMR in 2019 alone [2] and by 2050 this figure is predicted to grow to around 10 million [3, 4]. AMR occurs naturally but is accelerated by factors which *stress* microbes and cause them to evolve more quickly.

The most discussed driver of AMR is repeated human exposure to antimicrobial medicines [1]. This is closely followed by discussion of food producing animals, which contribute significantly to AMR as over 60% of global antimicrobial use is within the livestock sector, driving resistance and allowing AMR to spread through the food chain and into humans and the environment [5–7]. Other drivers of AMR include temperature changes, pollution (including from human and livestock waste) and changing host–pathogen dynamics [8, 9]. As microbes move through soils and water they are exposed to antimicrobial waste, pesticide residue and heavy metal pollution, all of which drive resistance by the same process as repeated exposure to antimicrobial medicines [5, 10]. Because AMR impacts upon human, animal, and environment health, we refer to it as a One Health challenge.

Many, if not all, drivers of AMR have a behavioural component. Thus, a growing number of research projects have begun to tackle AMR from a social perspective [11–15]. Antimicrobials in Society (AMIS) adopted an anthropogenic lens to understand human relationships with antimicrobials within the One Health sphere [16, 17]. The University of Oxford’s clinical research Unit in Vietnam is using a Randomized Control Trial (RCT) to assess the effectiveness of social interventions to create change on antimicrobial (mis)use in human and animal health [18]. Coordinated networks for behavioural AMR action are also appearing. Sonar Global was funded by Horizon 2020 to coordinate social science engagement within the response to AMR [19, 20] and in 2019 the University of Leeds established CE4AMR to support projects which address AMR via community engagement (CE) approaches.

The growing number of projects, collaborations, and networks exemplifies the importance and complexities of tackling AMR from behavioural and social science perspectives. Their outputs emphasize the imperative to develop equitable partnerships with whole communities, including gatekeepers, and wider stakeholders to address good stewardship around antimicrobials. When attempting to tackle the behavioural drivers of AMR, projects must engage the community within which they are working to support the development of context specific solutions. Messages and modes of communication must be bespoke and meaningful to the people the project wishes to work with, because behavioural changes can only be actioned by the people engaging in the behaviour.

## Main text

The CE4AMR network focuses on CE approaches to address AMR and has previously published methodological articles regarding what constitutes CE, and how CE can be equitably developed to address AMR [21, 22]. The network includes research projects such as the COSTAR collaboration, which aims to robustly evaluate the ability of the Community Dialogue Approach, or CDA [23] to tackle AMR in low resource settings. COSTAR builds upon five years of work to adapt the CDA to address AMR in Bangladesh [24] and uses knowledge gained from other CE approaches, in particular a Participatory Video (PV) project, to inform its content [25]. CE4AMR has also led the community co-development of an AMR educational resource pack with students and teachers in Nepal [26, 27], and hosted a recently completed PhD addressing the gendered dynamics of AMR behaviors [28]. All projects are summarized in Table 1 with links to publications and funding sources.

**Table 1:** An outline of recent CE4AMR projects which address AMR at community level in low-and-middle-income settings

Project name	Details	Setting	Gateway to research link	Publications
Sourcing Community Solutions to Antibiotic Resistance in Nepal (CARAN)	This project developed and pilot tested an interdisciplinary intervention to create community-level solutions to address the problem of antibiotic misuse in Nepal. A multi-disciplinary team trained participants in film making and supported them to develop videos to communicate the challenges they face regarding drug resistance. Films are now being used as advocacy tools to raise awareness of drug resistance within participants’ communities and to engage policy makers and ministry officials with community-level drug resistance challenges	Nepal	https://gtr.ukri.org/projects?ref=AH%2FR005869%2F1	[25, 28, 31]
Community dialogues for preventing and controlling antibiotic resistance in Bangladesh	This project adapted the Community Dialogue Approach (CDA) for addressing antibiotic resistance in Bangladesh. The CDA was piloted around five Community Clinics (which provide primary healthcare to around 30,000 people in total). Process evaluations determined its feasibility and acceptability to a range of stakeholders providing valuable learnings for how the CDA can be embedded into the existing health system and community infrastructure of Bangladesh	Bangladesh	https://gtr.ukri.org/projects?ref=ES%2FP004075%2F1	[24]
Developing community-led solutions to antimicrobial resistance: building a one health approach in low- and middle-income countries	This GCRF Challenge Cluster brought together six existing AMR research projects based across the Global South who use, or are interested in using, Community Engagement methods to tackle AMR. By consolidating learnings with those of local stakeholders in each country setting the cluster produced a handbook to guide the use of CE methods to tackle AMR in a variety of settings	Bangladesh, Ghana, India, Nepal, Vietnam	https://gtr.ukri.org/projects?ref=EP%2FT02335X%2F1	[21, 22, 30]
The use of creative arts to engage Nepali schools with antimicrobial-resistance and create positive behaviour change on health-seeking behaviours	This study co-created AMR educational materials with children and teachers based in Nepali secondary schools. The materials were then tested in both private and government funded schools, showing a significant increase in AMR knowledge of student’s post-test. Process evaluations also revealed that teachers and students benefited from the co-design process and enjoyed the flexibility of the teaching/learning materials to fit with teaching and learning styles and time scales	Nepal	https://gtr.ukri.org/projects?ref=AH%2FT007915%2F1	[21, 31]
Engaging communities to address antimicrobial resistance: Identifying contextualised and sustainable community-led solutions in low resource settings. (COSTAR)	The COSTAR study will combine PV and CDA methods to promote behaviour change on AMR in Nepal and Bangladesh. Multiple evaluative methods including a Randomised Control Trial (RCT) and process evaluations will be used to track the impact of the project from the perspectives of multiple stakeholders including community members, policy makers and health professionals	Bangladesh, Nepal	https://gtr.ukri.org/projects?ref=MR%2FT029676%2F1	
Using Participatory Video methods to explore gendered behaviors in AMR	This PhD chapter explored the gendered and One Health perspectives of AMR drivers through Community Engagement methods and advances our thinking of participatory video methodology in health research through a review and development of an interdisciplinary evaluation framework	Nepal	Forthcoming at http://www.ce4amr.leeds.ac.uk	[28]

All projects are based in LMICs, with in-country co-investigators and research partners designed to maintain long-term connections with community members, wider stakeholders, gatekeepers, and policy makers. This extensive engagement with Nepal and Bangladesh (many authors are locally based) has allowed CE4AMR team to unpick many of the complexities around communicating on AMR at community level and influencing meaningful behavioural change and actions [21, 25, 27, 28]. These can be distilled into seven key concepts that can be used to guide future CE for AMR interventions in low resource settings.

### CE4AMR key concepts for addressing AMR through behavior-change interventions.

#### Microbes are alive

This is the first point within many of the CE4AMR behavioural interventions. For communities to connect with the nuances of AMR an essential element within projects is to promote discussion about the often-novel concept of microscopic organisms (microbes) which cause illnesses. This allows reflections that many different microbes cause different illnesses, and that each different microbe will need a different treatment. The understanding of microbes as tiny living things also helps communities understand that microbes can move around in human and animal bodies and faeces, and in the soil and water. In some interventions, CE4AMR discusses ‘good’ microbes that help digest food, as this point helps clarify that taking antimicrobials when one is not sick could be harmful by killing off ‘good’ microbes.

#### Antimicrobials are very important medicines

The life-saving value of antimicrobial medicines is always emphasized within CE4AMR interventions. This is to prevent harm resulting from assumptions that to prevent AMR one should simply stop using antimicrobials. However, community members have reflected that saying ‘*antimicrobials save lives*’ may not always be contextually appropriate in communities where the timing and cause of death is considered predetermined by a higher power. Rather, CE4AMR emphasises that, when used properly, antimicrobials increase the quality of human and animal lives by maintaining health and productivity. The discussion can then move to how misusing antimicrobials can mean they do not work as effectively. This learning can be contextualised by exemplifying (via stories, visuals etc.) that there are many different types of illness and many types of medicines, including antimicrobials.

#### Seek health and veterinary professional advice before using antimicrobials

In all interventions the CE4AMR team stresses that communities should seek advice from a healthcare or veterinary professional to understand which medicine they or their animal should use and how. Doing so will mean that the appropriate medicine (maybe an antimicrobial) is used, and that the infection is fully treated. This is a challenging concept for many reasons. For example, in rural areas access to healthcare centres can be limited or geographically difficult, thus informal drug sellers and traditional healers are regularly consulted. In CE4AMR projects, this point often leads to discussion of finances because many communities believe that the antimicrobials available freely at their local level health facilities, such as health posts in Nepal, are less effective than more expensive options purchased for instance from pharmacies and drug-sellers. Affirming the expertise of health professionals, including vets, is an important message which can challenge this belief and increase engagement with accessible health services for humans and animals. Storytelling is a useful tool to exemplify this point by showing successful health outcomes of visiting government health facilities. That said, this is a point at which community engagement requires support from policy makers to strengthen infrastructure and allow intended behaviours to become actionable. One particular CE4AMR project used video making to share challenges around AMR behaviours with policy makers and highlight the reasons AMR-minimising behaviours are and cannot always be actioned [25].

#### There are repercussions of misusing antimicrobials

Point three is emphasized by discussing what could happen if antimicrobials are misused, for example via incorrect drug-bug matching or the taking of an incomplete dosage in humans and animals. In livestock and domesticated animals, antimicrobials are often misused to promote growth or prevent future disease (prophylaxis). In CE4AMR interventions, the discussion continues that if an incorrect antimicrobial medicine is taken to treat sick animals or humans, it may not kill the infection. Instead, this misuse can cause the infecting microbe to find ways to change to avoid the medicine, potentially leading to AMR. Similarly, if humans and animals do not complete a full course of antimicrobial treatment, the infecting microbe can survive. Indeed, a key driver of AMR is repeated exposure to low and incomplete doses of antimicrobials. CE4AMR finds that this message is well-received with regards to human, particularly child, health but can be more difficult in terms of animal health. This is primarily because veterinary professionals may not travel to rural areas, so there may be no other option but to buy antimicrobials without consultation (i.e., ‘over the counter’).

#### AMR happens to microbes

One of the most important points to communicate is that AMR happens to microbes, not to people or animals. This instils a greater sense of collective responsibility for addressing AMR. For example, individuals do not just need to be thinking about their own health-related behaviours, but also that of others. In terms of the COSTAR community dialogues, which aim to generate collective action and community driven solutions to AMR, this point is crucial.

#### Microbes, AMR, and antimicrobials can move around our environment

**Fig. 1:**
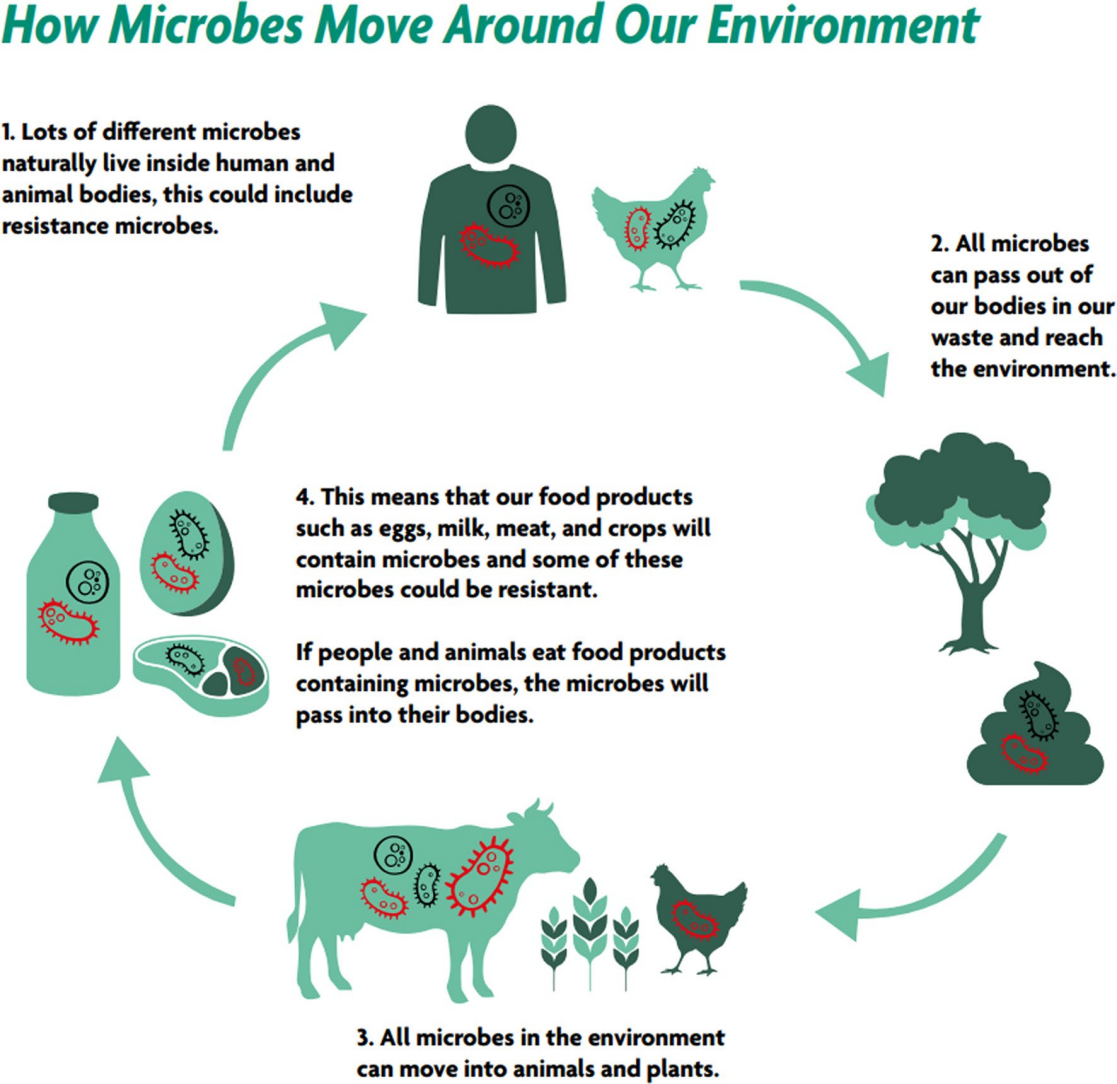
An example of the cycle diagram used in CE4AMR interventions (always contextually modified via iterative rounds of feedback with local stakeholders). This cycle demonstrates how microbes move around our environment between living things. It allows people to reflect upon drivers of AMR in their own lives and consider ways of breaking the chain of transmission

The concept of AMR happening to microbes helps people understand how AMR spreads. Activities can exemplify living microbes inside people, animals, water, and soil. This is important as it helps conceptualise the idea that microbes move around the environment, and that resistant microbes can also move. This can be difficult to understand without first thinking about microbes as living entities. The cycle diagram (Fig. 1) is often modified as a contextually appropriate drawing and used in CE4AMR-related activities, supporting community members to break the chain of transmission. For example, handwashing after touching animals or ensuring waste products do not go directly into the environment. When discussing the environment, it can help to use the terminology *our* environment to think about the local surroundings and impacts of AMR. An interactive community mapping exercise often helps people take a holistic view of how closely linked they, their families and animals are to their environment are, and crucially how they have the power to manage and reduce risks of cross contamination within this environment. Again, this can lead to collective action and meaningful local solutions.

**Fig. 2:**
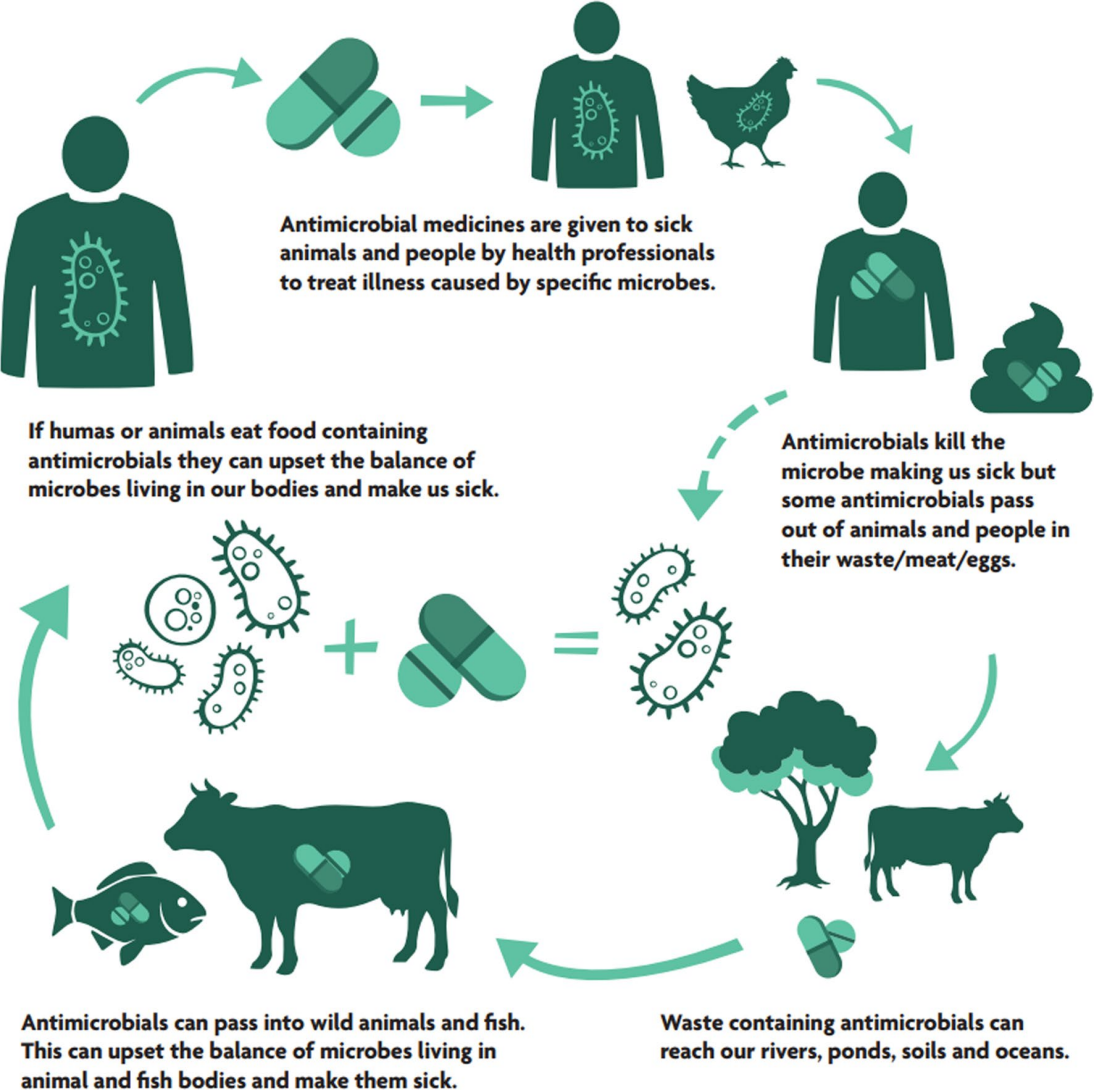
An example of the cycle diagram used in CE4AMR interventions (always contextually modified via iterative rounds of feedback with local stakeholders). This cycle focuses on the routes by which antimicrobials can move through our environments. It supports participants to consider AMR and antimicrobial use in their own lives and gives them a chance to explore ways of breaking the chain of transmission

Additionally, because antimicrobial medicines do not fully break down in human and animal bodies, they can pass out in waste and food products (e.g., milk), and can remain in meat products. This is important to explore with communities and CE4AMR often uses a second cycle diagram (Fig. 2) to demonstrate that antimicrobials do not end when they are taken as medicine. Communities can discuss ways of being careful with waste products and about consuming the meat, milk, and eggs of animals receiving antimicrobial treatment. CE4AMR activities attempt to link this point back to the messages around antimicrobials stressing microbes to develop resistance; if someone eats food containing antimicrobials, the microbes in their gut are likely to start finding ways to resist these medicines and develop resistance. The cycle diagrams are important in terms of visualising such dense information. They also highlight the need for joined-up and collective action on AMR within the One Health sphere. Communities can work together to identify AMR risks within their environments and ways they could minimise AMR developing and spreading.

#### Behavioural action against AMR requires more complex messaging than other global challenges

AMR is likened to other global challenges including the climate crisis [29] and water, sanitation and hygiene (WaSH) issues. Whilst the impacts of AMR in the long term could be similar, the immediate messaging around these issues differ. Within climate and WaSH messaging it is often appropriate to substitute a harmful behaviour with a safer behaviour. For example, rather than using a kerosene stove one could use a *clean-cook* stove to minimise emissions that contribute to global warming.

Messaging about cause and consequence in these contexts also tends to be linear: for example, washing hands with soap and water will ensure the removal of harmful microbes from the skin and prevent infection. However, when dealing with AMR, messages tend to be more complex. For example: *use antimicrobial medicines carefully to keep them working. If you use them too often and without a diagnosis, they may stop working and this means your illness would be harder to treat and could be more expensive*. This is an extremely detailed message with several points that need to be communicated before the conclusion can be realized. For this reason, whilst CE4AMR acknowledges interlinkages between AMR and wider global health challenges the nuances of AMR do require a bespoke approach to CE. CE4AMR projects use locally appropriate storytelling mechanisms within all their interventions because storytelling allows relatable narratives to be developed around AMR.

## Outlook

CE4AMR projects are ongoing, and the network continues to learn and adapt its approaches to tackle AMR at community level. AMR is a dense biological and social problem which requires diverse and interdisciplinary action if it is to be addressed properly at all levels of society. The community level is an appropriate space to deliver behavior-change interventions on AMR but the range of information to be communicated can be overwhelming. By sharing these seven *Key Concepts* and suggestions of how to apply them via CE, it is hoped CE4AMR’s storytelling approach may support other interventions that address AMR from a community, behavioural and social science perspective.

## Data Availability

Not applicable.

## Abbreviations

AMR: Antimicrobial resistance
CE: Community engagement
CE4AMR: Community engagement for antimicrobial resistance network
CDA: Community dialogue approach
PV: Participatory video
